# Population study of the gut microbiome: associations with diet, lifestyle, and cardiometabolic disease

**DOI:** 10.1186/s13073-021-01007-5

**Published:** 2021-12-17

**Authors:** Rebecca L. Walker, Hera Vlamakis, Jonathan Wei Jie Lee, Luke A. Besse, Vanessa Xanthakis, Ramachandran S. Vasan, Stanley Y. Shaw, Ramnik J. Xavier

**Affiliations:** 1 Broad Institute of MIT and Harvard, Cambridge, MA USA; 2 Center for Microbiome Informatics and Therapeutics, Massachusetts Institute of Technology, Cambridge, MA USA; 3 Department of Medicine, Yong Loo Lin School of Medicine, National University of Singapore, Singapore, Singapore; 4 Division of Gastroenterology and Hepatology, National University Health System, Singapore, Singapore; 5 Boston University and NHLBI’s Framingham Heart Study, Framingham, MA USA; 6 Department of Medicine, Section of Preventive Medicine and Epidemiology, Boston University School of Medicine, Boston, MA USA; 7 Department of Biostatistics, Boston University School of Public Health, Boston, MA USA; 8 Department of Medicine, Section of Cardiology, Boston University School of Medicine, Boston, MA USA; 9 Division of Cardiovascular Medicine, Brigham and Women’s Hospital, Harvard Medical School, Boston, MA USA; 10 Center for Computational and Integrative Biology, Department of Molecular Biology, Massachusetts General Hospital, Harvard Medical School, Boston, MA USA; 11 Klarman Cell Observatory, Broad Institute of MIT and Harvard, Cambridge, MA USA

**Keywords:** Framingham Heart Study, Gut microbiome, Cardiovascular disease, Type 2 diabetes, 16S rRNA gene sequencing, Microbial taxonomy

## Abstract

**Background:**

The human gut harbors trillions of microbes that play dynamic roles in health. While the microbiome contributes to many cardiometabolic traits by modulating host inflammation and metabolism, there is an incomplete understanding regarding the extent that and mechanisms by which individual microbes impact risk and development of cardiovascular disease (CVD). The Framingham Heart Study (FHS) is a multi-generational observational study following participants over decades to identify risk factors for CVD by correlating genetic and phenotypic factors with clinical outcomes. As a large-scale population-based cohort with extensive clinical phenotyping, FHS provides a rich landscape to explore the relationships between the gut microbiome and cardiometabolic traits.

**Methods:**

We performed 16S rRNA gene sequencing on stool from 1423 participants of the FHS Generation 3, OMNI2, and New Offspring Spouse cohorts. Data processing and taxonomic assignment were performed with the 16S bioBakery workflow using the UPARSE pipeline. We conducted statistical analyses to investigate trends in overall microbiome composition and diversity in relation to disease states and systematically examined taxonomic associations with a variety of clinical traits, disease phenotypes, clinical blood markers, and medications.

**Results:**

We demonstrate that overall microbial diversity decreases with increasing 10-year CVD risk and body mass index measures. We link lifestyle factors, especially diet and exercise, to microbial diversity. Our association analyses reveal both known and unreported microbial associations with CVD and diabetes, related prescription medications, as well as many anthropometric and blood test measurements. In particular, we observe a set of microbial species that demonstrate significant associations with CVD risk, metabolic syndrome, and type 2 diabetes as well as a number of shared associations between microbial species and cardiometabolic subphenotypes.

**Conclusions:**

The identification of significant microbial taxa associated with prevalent CVD and diabetes, as well as risk for developing CVD, adds to increasing evidence that the microbiome may contribute to CVD pathogenesis. Our findings support new hypothesis generation around shared microbe-mediated mechanisms that influence metabolic syndrome, diabetes, and CVD risk. Further investigation of the gut microbiomes of CVD patients in a targeted manner may elucidate microbial mechanisms with diagnostic and therapeutic implications.

**Supplementary Information:**

The online version contains supplementary material available at 10.1186/s13073-021-01007-5.

## Background

The gut microbiome’s direct contribution to health and disease status has been well established. From its extensive metabolic and synthetic capabilities and its intricate interactions with host immune system development and modulation, the microbiome greatly impacts host physiology [1]. Cohort-based studies of the microbiome have described substantial microbial variation even in the absence of disease [2]; established significant relationships between specific microbes and a variety of dietary factors, lifestyle factors, and anthropometric measurements [3, 4]; and demonstrated that microbial variability impacts inflammatory cytokine production capacity [5] and determines the level of immune education in early life [6], among other immunological processes.

Significant changes in microbial community composition and taxonomic abundances have been associated with different disease states across many organ systems [7–9]. These include cardiovascular and metabolic phenotypes such as obesity [10–12], type 2 diabetes (T2D) [13–17], and atherosclerotic cardiovascular disease [18–21] as well as metabolic risk factors for cardiovascular disease (CVD) such as hypertension [22, 23] and blood lipid levels [24, 25]. While cardiometabolic conditions are complex (with their etiology attributed to a combination of environmental, dietary, lifestyle, and genetic factors), the gut microbiome potentially provides a mechanistic link between many of these non-genetic factors and nutrient metabolism and inflammation in humans. A prominent example of this is the gut microbiota’s role in trimethylamine N-oxide (TMAO) production from dietary choline, betaine, and L-carnitine, coupled with studies implicating high levels of plasma TMAO in the risk of developing atherosclerosis [26–30]. Intestinal bacteria can also metabolize cholesterol to the biologically unavailable coprostanol, and the presence of these microbes in the gut microbiome is associated with decreases in both intestinal and serum cholesterol levels in multiple human cohorts [25]. Microbes also affect glycemic control, with specific taxa associated with higher blood glucose levels post-meal, explaining interindividual variability in postprandial response to identical meals [31]. The ability of the microbiome to exert multiple immune phenotypes [32] also suggests a role in the derangements of inflammation and innate and adaptive immunity implicated in atherosclerosis [33–35] and metabolic conditions such as obesity and T2D [36]. Taken together, clarifying microbial contributions to cardiometabolic conditions and related subphenotypes may provide mechanistic insights into disease and suggest preventive or therapeutic hypotheses based on modulating the microbiome.

The Framingham Heart Study (FHS) provides a unique opportunity to examine such relationships. Since its inception in 1948 as the first longitudinal, large-scale study of cardiovascular disease risk, the FHS has led to the identification of major risk factors for CVD such as smoking, obesity, high cholesterol, hypertension, physical inactivity, and diabetes [37]. The FHS cohorts studied in this report (Generation 3, Omni Generation 2, and New Offspring Spouse cohorts) represent a multi-ethnic, community-based sample of middle-aged men and women who have undergone genetic and phenotypic characterization.

Here, we present the first cross-sectional microbiome analysis of 1423 FHS cohort participants using 16S rRNA gene-based sequencing of stool samples to explore variation and function of the gut microbiome at the population level. Integrative analyses of lifestyle, dietary, and clinical factors with the microbiome reveal associations with overall microbial composition and diversity across participants. We identify microbial taxa and predicted functional pathways associated with a variety of clinical phenotypes, medications, and blood test measurements, providing insights into the molecular mechanisms by which microbiota influence health and cardiometabolic disease.

## Methods

### Cohort

For this study, FHS participants were aggregated from the Generation 3, OMNI 2, and New Offspring Spouse cohorts, three ongoing cohorts within the larger FHS study with examination cycles every 5–10 years. At Exam 3 (2016–2019), participants of all three sub-cohorts were informed of the microbiome study and given a stool sample kit with related questionnaire along with a variety of clinical tests, examinations, and health questionnaires. The actual participants of this study were those who returned a sample kit after the visit (*N*=1423). We generated 16S microbiome data from all 1423 participants using stool samples collected at Exam 3 of the FHS project. Stool samples were aliquoted upon receipt and stored until adequate batch sizes were met for extraction and 16S rRNA gene sequencing. To test variability caused by aliquoting and storage durations, 279 samples were extracted twice, for a total evaluation of 1702 samples.

FHS sub-cohort information is available at https://framinghamheartstudy.org/participants/participant-cohorts/ and summarized here. The sub-cohorts used in this study—Generation 3, OMNI 2, and New Offspring Spouse—reflect different enrollment criteria and efforts of the larger FHS research project. FHS originally recruited participants in their Original Cohort in 1948 by randomly sampling 2/3 of the adult population of Framingham, Massachusetts; this cohort was followed until 2014. The Offspring Cohort was initiated in 1971 consisting of offspring of the Original Cohort and their spouses; it was concluded in 2014. The Original Cohort and the Offspring Cohort were not a part of this study but used in describing participants of the sub-cohorts below:

- The Generation 3 Cohort represents the third generation of the Original Cohort (participants had at least one parent in the Offspring Cohort and were at least 20 years old by the close of the first exam cycle). Exam 1 began in 2002 and was completed in 2005. There were 4095 total participants in the Generation 3 Cohort.
- The New Offspring Cohort represents the spouses of an Offspring Cohort participant that were not previously enrolled and have at least two biological children who participated in Exam 1 of the Generation 3 Cohort. Recruitment started in 2003 and ended after Exam 1 in 2005. There were 103 total participants in the New Offspring Cohort.
- The OMNI 2 Cohort represents an effort to establish a group of participants that reflect diversity in the community. In 1994, an OMNI 1 Cohort was initiated and ended in 2014. To expand these efforts, enrollment for a second cohort of OMNI participants started in 2003 and ended in 2005. OMNI 2 included many individuals related to OMNI 1 participants as well as unrelated participants. There were 410 total participants in the OMNI 2 Cohort.

This study uses all sub-cohort participants providing a microbiome sample (Generation 3: *N*=1267; New Offspring: *N*=31; and OMNI 2: *N*=137) as one large dataset in all analyses described below.

### Metadata

#### Medications

Due to participants taking a wide variety of medications and many with similar mechanisms of action, we classified cardiovascular-related medications into 4 categories: antihypertensive agents, insulin and hypoglycemic medications, low-density lipoprotein (LDL)-lowering medications, and triglyceride (TG)-lowering medications. Antihypertensives included medications with FHS therapy group classification as “antihypertensives,” “agents acting on the renin-angiotensin system,” “beta blocking agents,” “calcium channel blockers,” and “diuretics.” Insulin and hypoglycemic medications are all drugs with FHS therapy group classification as “drugs used in diabetes,” which included insulin and its analogs and non-insulin blood glucose-lowering drugs such as metformin. LDL-lowering medications included medications with FHS chemical group classification as statins (“atorvastatin,” “lovastatin,” “pitavastatin,” “pravastatin,” “rosuvastatin,” and “simvastatin”), “alirocumab,” and “nicotinic acid.” Triglyceride-lowering medications included medications with FHS chemical group classification as “colesevelam,” “colestipol,” “cholestyramine,” “ezetimibe,” “fenofibrate,” “gemfibrozil,” and “omega-3 triglycerides.”

#### Diabetes status

Current diabetes status was defined as a study participant under current treatment for diabetes or with either a fasting blood glucose level of 126 mg/dL or greater or a non-fasting blood glucose level of 200 mg/dL or greater. Fasting status was defined as fasting for at least 8 h.

#### 10-year CVD risk

A 10-year risk for atherosclerotic cardiovascular disease was calculated for participants without a CVD diagnosis from age, sex, race, total cholesterol, HDL cholesterol, systolic blood pressure, blood pressure-lowering medication use, diabetes status, and smoking status based on a previously described model [38].

#### Metabolic syndrome

Metabolic syndrome status was defined as a study participant having 3 out of the 5 qualifications below, based off of the National Heart, Lung, and Blood Institute/American Heart Association guidelines [39].

1. Abdominal obesity: A waist circumference greater than or equal to 40 in. for men and greater than or equal to 35 in. for women.
2. High triglyceride levels: Triglycerides greater than or equal to 150 mg/dL or taking triglyceride-lowering drugs.
3. Low HDL levels: Less than 40 mg/dL for men and less than 50 mg/dL for women or taking LDL-lowering drugs.
4. High blood pressure: Either systolic blood pressure greater than 130, diastolic blood pressure greater than 85, or taking antihypertensives drugs.
5. High fasting blood sugar: Blood glucose levels greater than or equal to 110 mg/dL, calculated only for participants whose blood tests were performed during a fasted state.

#### Dietary recall

Dietary variables were taken from a questionnaire provided with the stool collection kit. Participants were asked to return a stool sample with a completed questionnaire of dietary intake within the last 7 days along with specific medication intake for the last 2 weeks. Medications include antibiotics, chemotherapy, and immunosuppressants. The dietary questionnaire gave a specific food category (“tea/coffee,” “sugary drinks,” “diet soft drinks,” “juice,” “water,” “alcohol,” “yogurt/foods with active bacterial cultures,” “dairy,” “probiotic,” “fruit,” “vegetables,” “beans,” “whole grains,” “starch,” “eggs,” “processed meat, ”, “red meat,” “white meat,” “shellfish,” “fish,” and “sweets”), and participants marked whether they did not consume the product in the past 7 days (0) or whether they consumed the product within the past 4 to 7 days (1), within the past 2 to 3 days (2), yesterday 1 to 2 times (3), or yesterday 3 or more times (4).

#### Physical activity

The physical activity questionnaire was given at Exam 3 and asked participants how many times per month they performed a specific activity. The activities surveyed included: “vigorous jogging or running,” “vigorous racket sports,” “bicycle,” “vigorous swimming,” “vigorous exercise class or dancing,” “vigorous activities: lifting, carrying, or digging,” “weights: snow shoveling, moving heavy objects, weight lifting,” “strenuous sports: basketball, football, skating, skiing, soccer, etc.,” “nonstrenuous sports,” “walk or hike,” “home exercise or calisthenics,” and “nonstrenuous weight training.”

### Sample handling and nucleic acid extraction

Stool samples were collected in 100% ethanol as previously described [7]. For DNA and RNA co-extraction, the QIAamp 96 PowerFecal Qiacube HT Kit (Qiagen Cat No./ID: 51531) was paired with the Allprep DNA/RNA 96 Kit (Qiagen Cat No./ID: 80311) and IRS solution (Qiagen Cat No./ID: 26000-50-2) for a custom protocol. For the initial lysis, 50–200 mg of stool per sample were transferred frozen into individual wells of the PowerBead plate, containing 0.1 mm glass beads (Cat No./ID: 27500-4-EP-BP), on a dry ice block. 650 μl of 55°C-heated PW1 buffer and 25 μL of freshly prepared 1M DTT were added directly to each sample well before lysis by bead beating on a TissueLyzer II at 20 Hz for a total of 10 min (in two 5-min intervals with plate rotation in between). Samples were pelleted by centrifugation for 6 min at 4500×*g*, and supernatants were transferred to a new S block (supplied in PowerFecal Kit), combined with 150 μl of IRS solution, and vortexed briefly before a 1-min incubation. Sealed samples were centrifuged again for 6 min at 4500×*g*, and up to 450 μl of supernatant were transferred to a new S block, combined with 600 μl of Buffer C4 (PowerFecal Kit), mixed by pipetting 10 times, and incubated for 1 min. Samples were transferred into AllPrep 96 DNA plates on top of clean S blocks and centrifuged for 3 min at 4500×*g*. The centrifugation step was repeated until all samples passed through. The AllPrep 96 DNA plate was stored at 4°C until ready for further processing.

The Allprep 96 DNA plate was removed from 4°C and placed on top of a 2-mL waste block. A 500 μl of AW1 buffer was added to the DNA plate and sealed prior to centrifugation for 4 min at 4500×*g*. The waste block was emptied after each wash step. A 500 μl of AW2 buffer was added to the DNA plate prior to sealing with AirPore tape and centrifugation for 10 min at 4500×*g*. The Allprep 96 DNA plate was placed on top of an elution plate. A 100 μl of 70°C-heated EB buffer was added to each sample column and incubated for 5 min. The DNA plate was sealed and then centrifuged for 4 min at 4500×*g* to elute, and DNA was stored at −20°C. All incubation and centrifugation steps were performed at room temperature.

### 16S rRNA gene sequencing

16S rRNA gene libraries targeting the V4 region of the 16S rRNA gene were prepared by first using qPCR to normalize template concentrations and determine optimal cycle number. Library construction was performed in quadruplicate with the primers 515F (5′-AATGATACGGCGACCACCGAGATCTACACTATGGTAATTGTGTGCCAGCMGCCGCGGTAA-3′) and unique reverse barcode primers from the Golay primer set [40, 41]. After amplification, sample replicates were pooled and cleaned via the Agencourt AMPure XP-PCR purification system. Prior to final pooling, purified libraries were normalized via qPCR in two 25 μL reactions, 2x iQ SYBR SUPERMix (Bio-Rad, REF: 1708880) with Read 1 (5′-TATGGTAATTGTGTGYCAGCMGCCGCGGTAA-3′) and Read 2 (5′-AGTCAGTCAGCCGGACTACNVGGGTWTCTAAT-3′) primers. Pools were quantified by Qubit (Life Technologies, Inc.) and sequenced on an Illumina MiSeq with 2 × 150 base pair reads using custom index 5′-ATTAGAWACCCBDGTAGTCCGGCTGACTGACT-3′ and custom Read 1 and Read 2 primers mentioned above.

### Operational taxonomic unit (OTU) construction and taxonomic assignment

OTU clustering and taxonomic profiling of 16S rRNA gene amplicon sequencing data were performed with the 16S bioBakery workflow built with AnADAMA2 [42], which incorporates ea-utils and the UPARSE pipeline (version 8.1). Briefly, paired-end reads from all datasets were first merged, filtered, and de-replicated. For quality-filtering the UPARSE threshold of Emax=1, at which the most probable number of base errors per read is zero for filtered reads, and a truncation quality threshold of 15 were used. Further reads were trimmed to a fixed length of 200 base pairs. OTUs were then sorted by size, singletons were discarded, and OTUs were clustered at 97% similarity. Subsequently, the representative sequences for each cluster were mapped against the Greengenes 16S rDNA database (version 13.5) to filter chimeras and obtain taxonomic assignment.

### Statistical analysis and visualizations

Analyses were performed in R (v 3.6.2) using the following packages: phyloseq (v1.30.0) [43], vegan (v2.5-7) [44], ape (v5.4-1) [45], microbiome (1.8.0) [46], MMUPHin (v1.0.0) [47], *q* value (v2.18.0) [48], ggplot (v3.3.3) [49], pheatmap (v1.0.12) [50], GGally (v2.1.0) [51], network (v1.16.1) [52, 53], and MaAsLin2 [54].

### Sample filtering and data quality control

The starting dataset consists of 1702 samples with 279 replicates (Additional file 1: Fig S1A). Replicates were first examined and showed high concordance, with an average Bray-Curtis distance between replicates of 0.1 and between non-replicates of 0.7 (Additional file 1: Fig S1B). Therefore, for samples with technical replicates, the replicate with the higher read depth was selected for downstream analysis. Samples were then required to have at least 15,000 mapped reads after OTU assignment (Additional file 1: Fig S1C). Data was converted to relative abundances, normalizing for library size. OTUs were filtered based on 5% prevalence, resulting in 1356 samples and 848 taxa, regardless of taxonomic classification level. Rarefaction curves indicated that read depth was sufficient to detect the majority of OTUs present in a sample, as the number of OTUs detected by read depth leveled off (Additional file 1: Fig S1D). Since samples were sequenced across 16 batches, the data were normalized for batch effects using MMUPHin, which takes as input a feature by sample matrix of microbial abundances, performs batch effect adjustment given provided batch information, and returns the batch-adjusted abundance matrix [47] (Additional file 1: Fig S1E). For the 848 identified OTUs, the most specific taxonomic assignment was available at the species level for 8.6% of OTUs, genus level for 34.9% of OTUs, family level for 41.7% of OTUs, and order level for 14.7% of OTUs (Additional file 1: Fig S1F).

### Diversity

Alpha-diversity [the number of observed OTUs (richness) and Shannon index (diversity)] and beta-diversity (Bray-Curtis dissimilarities) indices were calculated for all 1356 samples at the OTU level using the *phyloseq* package in R (version 1.30.0) [43]. We examined covariates of microbiome variation by calculating the association between FHS traits and community level ordination [non-metric multidimensional scaling (MDS) based on Bray-Curtis dissimilarities using *metaMDS*] with the *envfit* function from the R package *vegan* (version 2.5-6) [44]. We examined the effects of 25 variables: age, sex, HbA1c, ALT, AST, albumin, cholesterol, HDL, triglycerides, glucose, CRP, SBP, DBP, BMI, WHR, antibiotics, diabetes, CVD, CVD risk, metabolic syndrome, antihypertensives, LDL-lowering drugs, triglyceride-lowering drugs, insulin and hypoglycemic drugs, and Shannon diversity. To include CVD risk for all samples in this analysis, CVD risk for participants diagnosed with CVD was set to 1. *P* values were determined by 9999x permutations and adjusted for multiple testing of 25 traits using the Benjamini-Hochberg method. To examine how much variation could be explained by ordination, we also ran an MDS analysis using the *phyloseq* ordination function with method set to MDS (Additional file 1: Fig S2A). To examine which phylum-level taxonomic features contributed the most to variation across individuals, we performed pairwise Pearson correlations using the R function *cor* across the top 5 MDS axes, Shannon diversity, Simpson diversity, and all phylum-level relative abundances. The correlation matrix was visualized using the *corrplot* function [55] from the corrplot R package (Additional file 1: Fig S2B).

To assess variance explained in Shannon diversity from 50 environmental and lifestyle factors, we first regressed out the effects of age, sex, race, and antibiotic use on the Shannon diversity index for each sample by running a linear model and taking residuals. Then, for each of the 50 variables, we fit a linear regression model testing microbial diversity as a function of each variable to evaluate the variance in diversity explained by that variable. *P* values were corrected using Benjamini-Hochberg correction with significant associations identified at false discovery rate (FDR) <0.05. The 50 variables examined include: blood and anthropometric variables (HbA1c, ALT, AST, albumin, cholesterol, HDL, triglycerides, glucose, CRP, SBP, DBP, BMI, WHR, antihypertensives, LDL-lowering drugs, triglyceride-lowering drugs, and insulin and hypoglycemic drugs), dietary intake variables (tea/coffee, sugary drinks, alcohol, diet soft drinks, juice, water, yogurt/active bacterial cultures, dairy, probiotic, fruit, vegetables, beans, grains, starch, egg, processed meat, red meat, white meat, fish, shellfish, and sweets), and lifestyle variables measured as times per month (jog, racket sports, bike, swim, exercise class, vigorous activity, weights, strenuous sports, non-strenuous sports, walk, home exercise, and non-strenuous weights).

### Statistical association analyses

Association analyses were performed using an additive general linear model of OTU abundance for all 1356 participants as a function of sample metadata using MaAsLin2 [54]. To account for confounding effects, we included age, sex, race, and antibiotic use as fixed effects in the model in addition to the respective outcome variable. Statistical significance was corrected for multiple testing using the Storey-Tibshirani correction with significant associations identified at FDR<0.05. Associations with CVD risk were only performed on participants without a history of CVD (*N*=1294). For the analysis of anthropometric and blood analyte measurements, we examined 13 variables: HbA1c, ALT, AST, albumin, cholesterol, HDL, triglycerides, glucose, CRP, SBP, DBP, BMI, and WHR. For the analysis of diagnostic conditions and selected medications, we examined 27 variables: cardiometabolic traits (CVD, CVD risk, diabetes, metabolic syndrome, aspirin, antihypertensives, LDL-lowering drugs, triglyceride-lowering drugs, and insulin and hypoglycemic drugs), diagnostic variables that were at least 5% prevalent in the cohort (cancer, migraine, thyroid disease, gynecological problems, asthma, GERD, depression, and anxiety), and drugs related to these diagnostic variables with FHS therapy groups (analgesics, antidepressants, anxiolytics, thyroid therapy, hormonal contraceptives, progestogens, estrogens, antimigraine preparations, drugs for acid related disorders, and drugs for obstructive airway diseases).

### Correlation network graphs

To visualize correlations among associations for specific variables, all significant associated OTUs (FDR<0.05) for the given variables determined by MaAsLin2 [54] were plotted as nodes in the network with edges being defined by Spearman’s correlation between the OTU and given variables. The network was constructed using the *ggnet2* function from the *GGally* R package [51].

### Functional profiling

PICRUSt software was used to predict the metagenome functional potential based on 16S marker genes [56]. First, OTU abundances were normalized by their known or predicted 16S copy number. To reduce irrelevant terms, KEGG pathway-level categories were summarized and required to contain any of the following keywords: “metabolism,” “synthesis,” “pathway,” “degeneration,” or “bacterial.” We additionally removed terms specific to fungi (*N*=142). Pathway-level categories were converted to relative abundances to normalize library size and filtered for prevalence of at least 10%, resulting in 134 pathways. Association analysis was performed on all 1356 samples using MaAsLin2 [54], correcting for covariates of age, sex, race, and antibiotic use. Statistical significance was corrected for multiple testing using Storey-Tibshirani correction with significant associations identified at FDR<0.05. Associations with CVD risk were only performed for participants without prevalent clinical CVD.

## Results

### Study population and participant characteristics

**Fig. 1:**
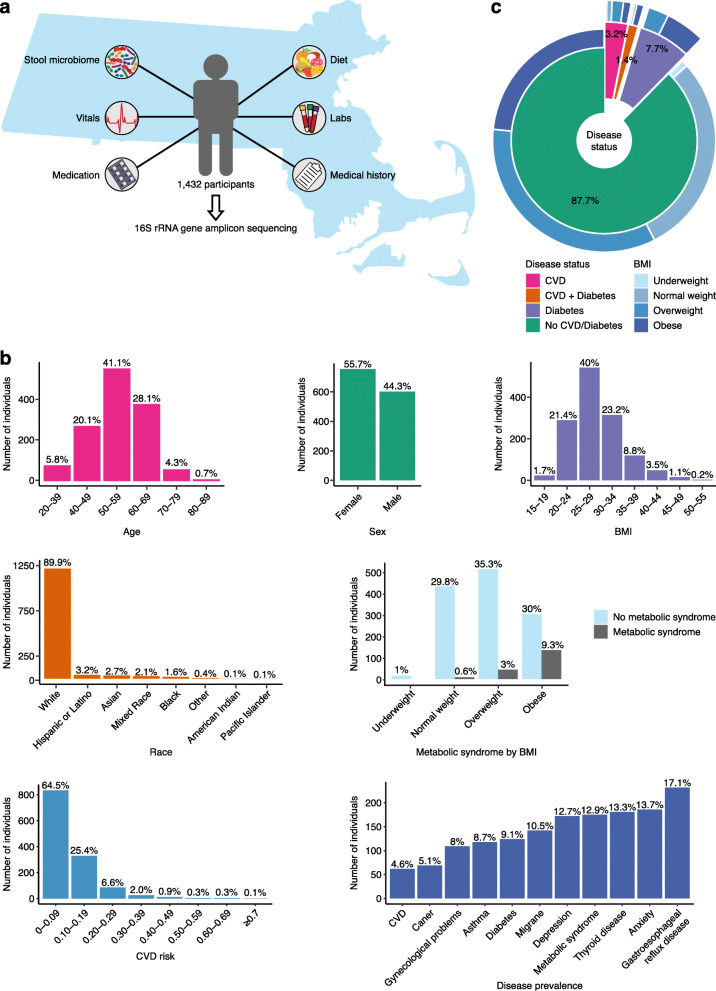
Summary of data types and demographics of FHS. **a** Illustration of our experimental design and data profiles per participant. **b** Histograms of demographics of the cohort, for age, sex, BMI, race, metabolic syndrome as it relates to BMI distribution, CVD risk, and disease prevalence. **c** Distribution of CVD and diabetes in the cohort, with BMI distributions per diagnostic category

From 2016 through 2019, FHS participants underwent an extensive third examination cycle consisting of stool sample collection for microbiome profiling along with numerous clinical interviews, physical examination, laboratory tests, non-invasive tests, and health questionnaires (Fig. 1A). 16S rRNA gene amplicon sequencing was performed on 1702 stool samples from 1423 participants, including 279 technical replicates (Additional file 1: Fig S1A). Technical replicates allowed for the examination of variability caused by aliquoting and storage durations during the collection time period. Data for replicates were generated with a gap of 4 to 20 months, during which time samples were stored at −80°C. Similarity analysis showed high concordance with an average Bray-Curtis distance of 0.1, where the average Bray-Curtis distance between non-replicate samples was 0.7 (Additional file 1: Fig S1B). This supports the validity and robustness of our sample processing methods. After selecting the replicate with the higher read depth and downstream quality control (Methods, Additional file 1: Fig S1A), the data consisted of 1356 participants. The demographics of these participants were notable for 55.7% female, 89.9% self-reported race “white,” average age of 55 years (range 32–89 years), and average BMI of 28 kg/m^2^ (range 15–51 kg/m^2^) (Fig. 1B). All participants completed questionnaires surveying medical history, lifestyle and dietary recall, and prescription and non-prescription medications; underwent laboratory exams of blood specimens; and reported hospitalizations or cardiovascular events that were subjected to clinician review and adjudication. Among study participants, the rates of CVD (4.6%), T2D (9.1%), metabolic syndrome (18%), and obesity (30%) were slightly lower than the prevalence among adults in the USA (Fig. 1C) [57].

### Microbial diversity correlations with participant phenotypes

**Fig. 2:**
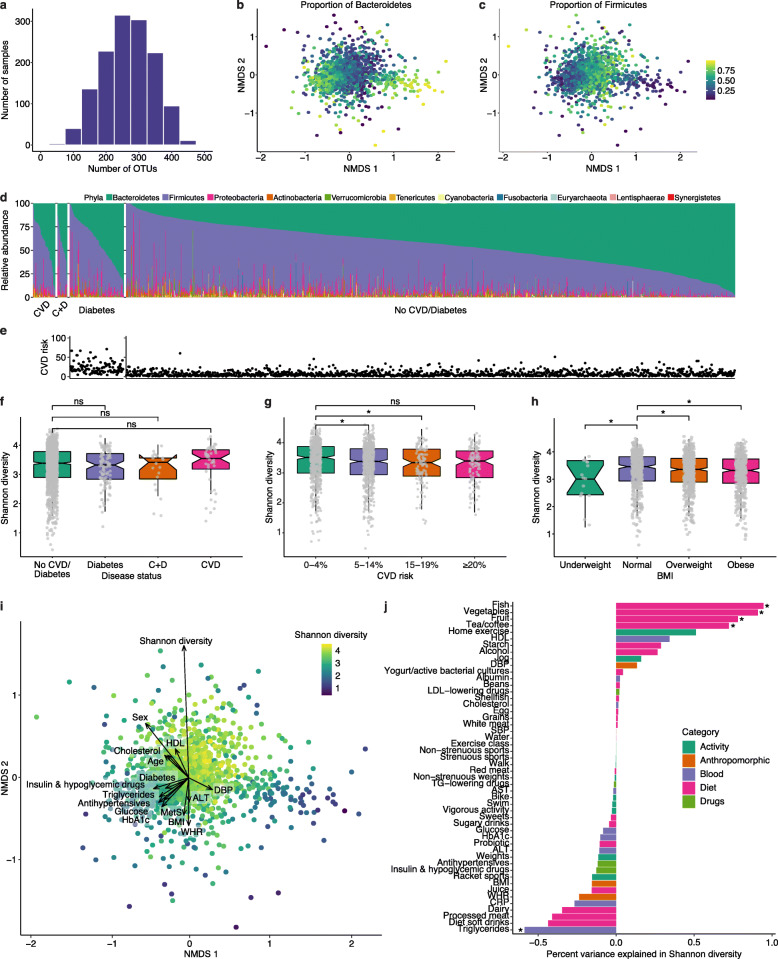
Compositional diversity of the gut microbiome. **a** Histogram of alpha diversity. Richness of each sample is shown as the number of OTUs detected per sample. **b**, **c** Non-metric multidimensional scaling (NMDS) analysis of Bray-Curtis distances calculated from OTU-level relative abundances. NMDS 1 and 2 are shown on the *x*- and *y*-axis, respectively. Each dot represents an individual and is colored by the proportion of Bacteroidetes (**b**) or Firmicutes (**c**) abundance. **d** Overall phylum-level relative abundance composition across all samples binned by cardiometabolic disease status of CVD, CVD plus diabetes (denoted C+D), diabetes, and no CVD or diabetes. Each sample is represented by one stacked bar colored by phylum. **e** A 10-year CVD risk score calculated on all participants without a CVD diagnosis. Each dot represents an individual that corresponds to a bar in **d** and indicates risk for developing CVD as a probability from 0 to 100%. **f** Boxplots of Shannon diversity across cardiometabolic disease status. Wilcoxon test comparing each disease status to no CVD or diabetes found no significant (ns) difference between Shannon diversity across these categories. **g** Boxplots of Shannon diversity across binned 10-year CVD risk scores. Wilcoxon test comparing 0–4 to 5–14% and 15–19% CVD risk are significant (* indicates *p*<0.05). **h** Boxplots of Shannon diversity across BMI classification. Wilcox test comparing normal BMI to all other BMI categories are significant (* indicates *p*<0.05). **i** NMDS analysis of Bray-Curtis distances calculated from OTU-level relative abundances. NMDS 1 and 2 are shown on the *x*- and *y*-axis, respectively. Each dot represents an individual and is colored by Shannon diversity. Factors were fit onto this ordination by the *envfit* function in R. Factors that significantly correlated with vector projections in the ordination space are shown. The strength of the correlation is depicted by the length of the arrow, which points in the direction of the variable that changes most rapidly and with maximum correlation with the ordination configuration. **j** A bar plot of percent variance explained in Shannon diversity (*x*-axis) explained by each variable (*y*-axis). Each variable is colored by its corresponding category. Each variable was tested for association with Shannon diversity by fitting a linear model, and the percent variance explained represents the (sign of the estimate) (*r*^2^)*100. Asterisks indicate significance at corrected *p*<0.05

We examined microbial composition based on 16S rRNA gene amplicon sequencing by first assessing richness and alpha and beta diversity indexes. The observed richness of the cohort, assessed as the number of microbial OTUs detected per participant, was on average 267 OTUs, ranging from 32 to 474 (Fig. 2A). While the majority of samples had a large number of taxa represented, the evenness of the communities varied greatly between individuals. The relative abundance of Bacteroidetes ranged from 0 to 98%, while the relative abundance of Firmicutes ranged from 2 to 99%, with the Shannon diversity index strongly correlated to Firmicutes (Pearson’s correlation *r*=0.71, *p*<2.2e−16) and negatively correlated to Bacteroidetes (Pearson’s correlation *r*=−0.69, *p*<2.2e−16). We used Bray-Curtis distance to calculate beta diversity and observed that the proportion of Bacteroidetes and Firmicutes was the largest driver of variation across individuals observed for taxonomic features at the phylum level, as visualized by non-metric multidimensional scaling (NMDS; Fig. 2B, C, Additional file 1: Fig S2A, B). Complete phyla relative abundances showed contributions from Proteobacteria to be high in a few samples ranging from 0 to 89% but with an average relative abundance around 4% (Fig. 2D). Among the samples with high Shannon diversity (>4), the relative abundances of Bacteroidetes and Firmicutes were 27% and 64%, respectively.

We calculated an estimate of 10-year risk for atherosclerotic CVD for all participants without prevalent CVD based on CVD-related factors including total and high-density lipoprotein (HDL) cholesterol, systolic blood pressure, medications for blood pressure, diabetes status, and smoking status (Fig. 2E). While there was no statistically significant difference between Shannon diversity among individuals with prevalent CVD and diabetes compared to the rest of the cohort (Fig. 2F), we did find significant differences in diversity measures by CVD risk (Fig. 2G). Additionally, consistent with previous reports [58], we observed decreased Shannon diversity with increased BMI status (Fig. 2H).

To assess contributions of measured phenotypic variables on overall microbiome composition, we examined anthropometric and blood test measurements as well as medications related to managing diabetes and heart disease, along with CVD, diabetes, CVD risk, and metabolic syndrome status. Each variable was fit onto an ordination plot to identify which variable changed in correlation to the ordination configuration (Fig. 2I). While Shannon diversity showed the strongest correlation (*r*^2^=0.0931, FDR=0.0005), there were significant correlations to the ordination plot with 16 additional variables, though to a lesser degree (Additional file 2: Table S1). Related variables such as triglycerides, glucose, HbA1c, BMI, WHR, antihypertensives, insulin and hypoglycemic drugs, diabetes, CVD risk, and metabolic syndrome showed similarities in strength of correlation and directionality in the ordination plot, suggesting these associations may be driven by a unifying factor.

Next, we wanted to assess the contribution of environmental and lifestyle factors on microbiome alpha diversity. We analyzed 50 variables from the dietary recall and physical activity questionnaires, anthropometric and blood analyte measurements, and diabetic and cardiovascular medications to test for associations with Shannon diversity, while correcting for age, sex, race, and antibiotic use. Dietary factors showed the strongest associations with diversity; frequency of consuming fish, vegetables, fruit, tea, and coffee within the last week were positively associated with diversity. Blood triglyceride concentration was the only significant variable negatively associated with diversity. Factors with trending significance (*p*<0.05, FDR>0.05) included leisure-time exercise, HDL cholesterol, and starch consumption, which were positively associated with microbial diversity, and intake of diet soft drinks, processed meat, and dairy, which were inversely associated with Shannon diversity (Fig. 2J, Additional file 2: Table S2).

### Associations of anthropometric and blood analyte measurements with the microbiome

**Fig. 3:**
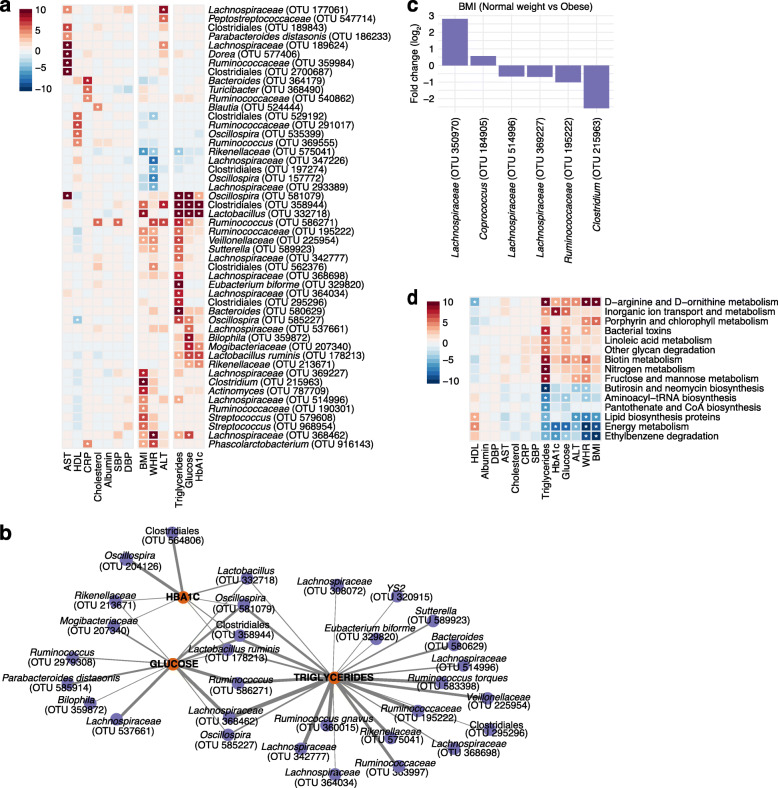
Significant microbial and functional associations of blood test and anthropomorphic measurements. **a** A heatmap depicting the top 50 associations across 13 blood test and anthropomorphic measurements colored by the -log(*q* value)*sign(coefficient). Significant associations (corrected *p*<0.05) are indicated by an asterisk. **b** Network of significant associations between microbial taxa (purple circles) and triglycerides, glucose, and HbA1c (orange circles). The thickness of edge (gray) is defined by the strength of Spearman correlation (r) between the relative abundance of the OTU and each feature; heavier edge weight implies stronger correlation. **c** Bar plot of the log fold change of the average relative abundance for significant OTUs associated with BMI status of normal weight vs obese participants. A positive fold change corresponds to OTUs with greater abundance in participants of normal weight, and a negative fold change corresponds to those with greater abundance in participants who are obese. **d** A heatmap depicting the top 15 functional pathway associations predicted from PICRUSt across 13 blood test and anthropomorphic measurements colored by the -log(*q* value)*sign(coefficient). Significant associations (corrected *p*<0.05) are indicated by an asterisk

To identify relationships between abundance of individual microbial taxa with population-wide phenotypic measurements, we related microbial OTUs to 13 anthropometric and blood analyte measurements. After FDR correction and adjusting for age, sex, race, and antibiotic use, we detected 129 statistically significant associations, identifying 89 taxonomic units across 11 phenotypic measurements (Fig. 3A, Additional file 2: Table S3). We observed two clusters of host phenotypic measurements in which multiple phenotypes displayed similar patterns of OTU associations: (1) BMI with WHR and (2) blood analyte measurements related to triglyceride and glucose phenotypes.

The phenotypic measurements with the most microbial associations were WHR, followed by blood triglycerides and BMI. WHR and BMI displayed strong overlapping associations with increases in OTUs representing *Lachnospiraceae*, *Ruminococcaceae*, *Veillonellaceae*, and *Phascolarctobacterium* and decreases in *Rikenellaceae*. Triglycerides, glucose, and HbA1c were associated with increases in a nexus of OTUs that included Clostridiales, *Lactobacillus*, *Oscillospira*, *Lactobacillus ruminis*, *Ruminococcus*, *Lachnospiraceae*, *Rikenellaceae*, and *Mogibacteriaceae* (Fig. 3B). Four OTUs were significant across all three of these blood markers, and five OTUs were significant among two of the three measurements. Each had unique associations as well.

BMI is a measure that has been widely examined in relation to the gut microbiome. We identified a total of 22 OTUs associated with BMI. To further explore this relationship, we compared participants with BMI classified as normal weight (18.5–25 kg/m^2^) and obese (≥30 kg/m^2^) and evaluated a categorical-based association analysis. Two OTUs (*Lachnospiraceae* and *Coprococcus*) were increased among participants of normal weight, and four OTUs (two *Lachnospiraceae*, *Ruminococcaceae*, and *Clostridium*) were enriched in obese participants (Fig 3C, Additional file 1: Fig S3A).

We then assessed the functional capabilities of the implicated microbial communities by inferring the metabolic potential of associated taxa from predicted metagenomic content of 16S rRNA marker genes using PICRUSt [56]. We identified 130 statistically significant associations among 64 KEGG pathway-level categories for nine of the anthropometric and blood analyte measurements (Additional file 2: Table S4). While many of the significant categories were non-discriminative and functions of all bacteria, we observed similar enrichment patterns among related phenotypic features such as BMI, WHR, ALT, glucose, HbA1c, and triglycerides. Among these top associations, we found positive correlations with D-arginine and D-ornithine metabolism and biotin metabolism as well as negative correlations with ethylbenzene degradation across the most features (Fig. 3D).

### Disease diagnoses and medication associations with the microbiome

**Fig. 4:**
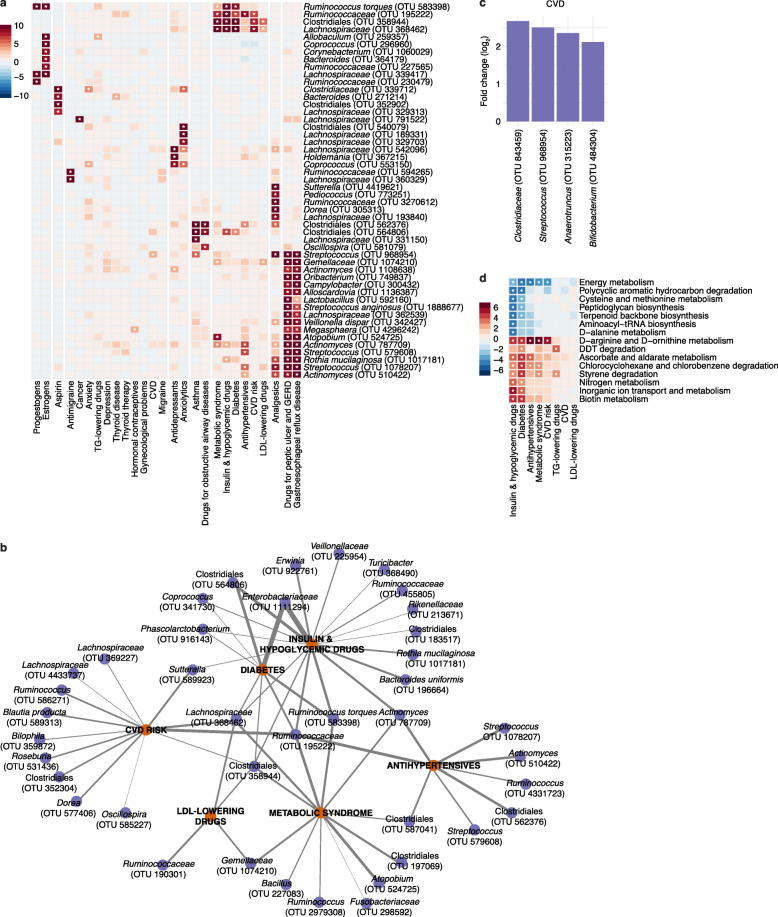
Significant microbial and functional associations of disease diagnostics and medication intake. **a** A heatmap depicting the top 50 associations across 27 disease diagnostics and medications colored by the -log(*q* value)*sign(coefficient). Significant associations (corrected *p*<0.05) are indicated by an asterisk. **b** Network of significant associations between microbial taxa (purple circles) and CVD risk, diabetes, metabolic syndrome, antihypertensives, LDL-lowering drugs, and insulin and hypoglycemic drugs (orange circles). The thickness of edge (gray) is defined by the strength of Spearman correlation (*r*) between the relative abundance of the OTUs and each feature; heavier edge weight implies stronger correlation. **c** Bar plot of the log fold change of the average relative abundance for significant OTUs associated with CVD status. **d** A heatmap depicting the top 15 functional pathway associations predicted from PICRUSt across 8 cardiometabolic diagnostics and medications colored by the -log(*q* value)*sign(coefficient). Significant associations (corrected *p*<0.05) are indicated by an asterisk

To explore the relationship between disease risk and the microbiome, we assembled a list of 27 diagnostic conditions and selected medication variables. These included CVD classification based on clinician-verified cardiovascular events, a derived classification of diabetes (based on fasting blood glucose levels and treatment for diabetes), the FHS 10-year CVD risk score (Methods), a derived classification of metabolic syndrome (Methods), questionnaire-based diagnoses for non-CVD and non-diabetes conditions with at least 5% prevalence in the FHS cohort, and corresponding medications for the conditions examined. After FDR correction and adjusting for age, sex, race, and antibiotic use, we detected 281 statistically significant associations, identifying 848 taxonomic units across 26 phenotypic measurements (Fig. 4A, Additional file 2: Table S5).

The two variables representing gastrointestinal phenotypes (gastroesophageal reflux disease [GERD] and drugs for peptic ulcers and GERD) had the most associations, with 24 and 27 OTUs respectively, followed by anxiolytics (anti-anxiety drugs) with 27, analgesics (pain killers) with 23, estrogens with 21, and insulin and hypoglycemic drugs with 18 associated OTUs. Interestingly, variables with the most OTU associations were often oral medications that directly interact with the gut, whereas diagnosis variables for the corresponding conditions did not exhibit as many associations. For example, participants with diabetes were defined as those who either (1) were taking insulin or/and hypoglycemic drugs, (2) had a fasting blood glucose level of 126 mg/dL or greater, or (3) had a non-fasting blood glucose level of 200 mg/dL or greater. This group, therefore, included more than just individuals taking medication for diabetes. All eight microbes associated with a diabetes diagnosis were also significantly associated with insulin and hypoglycemic drugs (OTUs representing *Enterobacteriaceae*, *Ruminococcaceae*, *Coprococcus*, two Clostridiales, *Lachnospiraceae*, *Ruminococcus torques*, and *Phascolarctobacterium*), although the medication variable showed stronger statistical significance and identified additional microbial associations (Fig. 4B).

Of the eight associations with diabetes, three OTUs representing *Ruminococcaceae*, Clostridiales, and *Lachnospiraceae* were also significantly associated with CVD risk and metabolic syndrome (Fig. 4B). These three OTUs were also associated with BMI, blood triglycerides, and glucose levels (Fig. 3A), with additional overlaps for a subset of the OTUs with antihypertensives or LDL-lowering drug associations (Fig. 4B). However, the four OTUs associated with prevalent CVD did not overlap with those associated with related diagnostic and medication intake (diabetes, insulin and hypoglycemic drugs, CVD risk, and LDL-lowering drugs) (Fig. 4C, Additional file 1: Fig S3B). One of the four OTUs, *Streptococcus*, was associated with BMI and non-cardiovascular-related variables including analgesics, asthma, GERD, and drugs for peptic ulcers and GERD.

We again inferred the functional potential of the microbial communities using PICRUSt [56] and assessed how this corresponded with CVD- and diabetes-related variables. We identified 88 significant associations among 42 KEGG pathway-level categories for seven of eight cardiovascular-related variables: diabetes, drugs for diabetes, triglyceride-lowering drugs, LDL-lowering drugs, CVD risk, metabolic syndrome, and antihypertensives but not CVD (Additional file 2: Table S6). Diabetes and drugs for diabetes showed the strongest signal with 25 and 30 significant pathway associations, respectively. We observed similar pathway-level categories significant across cardiovascular-related variables as those associated with anthropometric and blood analyte measurements, with related variables showing more overlap. For example, the positive association with chlorocyclohexane and chlorobenzene degradation that was observed with diabetes, insulin and hypoglycemic drugs, antihypertensives, and metabolic syndrome was also observed with HbA1c (Fig. 4D).

## Discussion

The extensive clinical profiling of the Framingham Heart Study, along with its large sample size and variety of data types collected on each participant, provides a unique opportunity to characterize the gut microbiome as it relates to cardiovascular and metabolic disease phenotypes. We utilized 16S rRNA gene sequencing to identify significant microbial associations with phenotypes including blood laboratory analyte measurements, anthropometric measurements, medications, and prevalent disease. We observed no phylum-level compositional changes among participants with prevalent CVD and diabetes relative to the rest of the cohort. Our analyses (adjusted for age, sex, race, and antibiotic use) revealed that OTUs representing *Ruminococcaceae*, Clostridiales, and *Lachnospiraceae* were significantly associated with CVD risk, diabetes, and metabolic syndrome, as well as medications such as lipid-lowering, hypoglycemic and antihypertensive agents (Fig. 4B), and cardiometabolic phenotypes such as BMI, triglycerides, and glucose (Fig. 3A). A complementary analysis revealed that several cardiometabolic phenotypes showed similar strength of correlation and directionality in an ordination plot (Fig. 2I) including CVD risk, diabetes, and metabolic syndrome; hypoglycemic and antihypertensive medications; and BMI, triglycerides, glucose, and HbA1c. While this cross-sectional analysis of microbial associations is not causal, these findings suggest that microbial-mediated mechanisms could contribute to CVD risk and the cardiometabolic phenotypes that comprise metabolic syndrome through shared mechanisms.

In addition, microbial diversity significantly decreased with increasing a 10-year CVD risk as well as with increasing BMI measures. When examining factors contributing to microbial diversity, dietary factors of fish, vegetables, fruit, tea, and coffee together explained 3.3% of variance in Shannon diversity, highlighting the impact of diet on the microbiome. Previous studies of the impact of dietary factors on the gut microbiome identified the same food groups exhibiting similar effects on the microbiome [4, 59]. These include food groups we found associated with increased Shannon diversity (nuts, fish, fruits, vegetables, and cereals/grains) exhibiting similar microbial associations with bacteria known to be anti-inflammatory through short-chain fatty acid production as well as food groups we found associated with decreased Shannon diversity (processed meats, soft drinks, and sugar) associating with three Firmicutes species implicated in obesity [59, 60], which we also correlated with decreased microbial diversity. Further examination of dietary intake at the macronutrient level may provide increased statistical power to detect diet-induced microbial changes, as diet has a dominant and rapid influence on microbiome composition [3, 61].

Given the wide variety of factors available through clinical interviews, physical examinations, laboratory assays, and health questionnaires cohort-wide, we identified strong statistical associations with many risk factors for cardiometabolic diseases, such as BMI, triglycerides, blood glucose, and HbA1c. Numerous OTUs of the order Clostridiales, including family classifications of *Ruminococcaceae*, *Lachnospiraceae*, and *Rikenellaceae*, associated with both BMI and triglycerides, have been previously reported in multiple studies of 16S and metagenomic data [3, 14, 24]. In addition, an increase in *Lactobacillus* abundance with HbA1c and glucose levels in the blood has been consistently reported [62, 63].

We observed the strongest microbial signal for cardiometabolic diagnoses and corresponding drugs for insulin and hypoglycemic drugs, with 18 associations, followed by diabetes with eight associations. All eight of the diabetes-associated OTUs were also significantly associated with insulin and hypoglycemic drugs, suggesting that drug intake may influence the diabetes signal, as the microbiome is known to interact with pharmaceuticals [64]. Metformin, a common anti-diabetic medication, exhibits a strong effect on the gut microbiome; its use is associated with increased *Akkermansia muciniphila*, *Escherichia coli*, and *Bifidobacterium bifidum* [65–67]. While these species were not associated with insulin and hypoglycemic drugs in our analysis, we did not specifically examine effects of metformin alone. Participants using metformin in the FHS cohort took on average three additional drugs to treat cardiometabolic disease, including statins and antihypertensives, which also interact with gut microbiota [3, 68]. However, we associated *Coprococcus* with both diabetes status and insulin and hypoglycemic drugs; this genus has been previously associated with metformin intake in the LifeLines-DEEP population-based metagenomic study [3]. The LifeLines-DEEP study also reports multiple *Lachnospiraceae*, *Ruminococcaceae*, and Clostridiales species associated with statins intake, which were associated with all LDL-lowering drugs in our cohort [3]. Of the four taxonomic associations with CVD that we identified, an increase of *Streptococcus* was previously reported in a metagenomic study of atherosclerotic CVD patients [18], while *Bifidobacterium* and *Clostridiaceae* were both associated with history of myocardial infarction in the LifeLines-DEEP study [3]. Given the heterogeneity in the overall microbial composition observed in participants with prevalent CVD and diabetes, further participant recruitment targeted for these diseases would provide statistical power to detect additional connections between gut microbiota and disease pathogenesis.

Large, community-based population studies of the microbiome have the potential to increase our understanding of how the microbiome may be a mechanistic mediator of environmental, dietary, lifestyle, clinical, and genetic factors that are associated with susceptibility to cardiovascular and metabolic diseases. Previous work on microbial cholesterol metabolism and TMAO production has demonstrated the utility of further interrogating microbes identified by association studies, as this work revealed mechanistic insight into how microbes impact host biology [25, 27, 29]. While large cohorts with multiple prevalent diseases have advantages compared to smaller case–control studies for single conditions, complexity is also increased in community- and population-based studies. For instance, we did not observe previously reported microbial associations with metformin, which may be confounded by comorbidities and the use of multiple medications. Furthermore, it remains challenging to disambiguate the potential effects on the microbiome of a disease process (e.g., type 2 diabetes) from the effects of medications that are taken to treat the disease or reduce risk.

The data generated in this study will provide the translational research community a valuable resource to leverage for further examining the role of the microbiome in the many other disease conditions and clinical variables collected at FHS. Despite the large sample size of our cohort, its rich phenotyping and long-term follow-up, study designs with greater racial and ethnic diversity, sequential longitudinal stool sample collection, integration of host genetic data, more detailed molecular stool and blood assessments, and deeper metagenomic sequencing will enable more mechanistic hypotheses to be generated in the future.

## Conclusions

We demonstrated that the gut microbiome is associated with a variety of cardiometabolic phenotypes, with the identification of both novel and previously reported associations between microbes and risk factors for disease diagnoses, disease development, and pharmaceutical intake. We found that overall gut diversity changes were associated with increased risk for developing CVD and increased BMI status, and we also showed that diversity is influenced most by dietary factors. We further identified a set of microbial OTUs that overlap in their associations with CVD risk, metabolic syndrome and diabetes, enabling hypothesis generation regarding shared microbial mechanisms underlying metabolic syndrome, diabetes, and CVD. This sets the stage to further explore how microbiome heterogeneity plays a role in the response to dietary and lifestyle factors for cardiometabolic disorders. In addition, higher resolution taxonomic characterization of the microbiome by metagenomic sequencing and complimentary data types such as metabolomics, as well as longitudinal assaying of participants with increased risk for CVD and integration of host genetic data, will allow for further identification and characterization of microbial factors involved in the pathogenesis of CVD and diabetes.

## Supplementary Information

**Additional file 1.** Supplementary figures. This file (.pdf) contains Figures S1-S3.

**Additional file 2.** Supplementary tables. This file (.xlsx) contains Tables S1-S6.

## Data Availability

The code to reproduce all the figures in this study is available at https://gitlab.com/xavier-lab-computation/public/fhs-16s [69]. The datasets generated and/or analyzed in this study are available in the Sequence Read Archive (SRA, PRJNA758252) at https://www.ncbi.nlm.nih.gov/bioproject/PRJNA758252 [70]. Correspondence and requests for materials should be addressed to the Framingham Heart Study at https://framinghamheartstudy.org/fhs-for-researchers/ (R.S.V.).

## Abbreviations

ALT: Alanine transaminase
AST: Aspartate aminotransferase
BMI: Body mass index
CRP: C-reactive protein
CVD: Cardiovascular disease
DBP: Diastolic blood pressure
FDR: False discovery rate
FHS: Framingham Heart Study
GERD: Gastroesophageal reflux disease
HbA1c: Glycated hemoglobin
HDL: High-density lipoprotein
LDL: Low-density lipoprotein
NMDS: Non-metric multidimensional scaling
OTU: Operational taxonomic unit
SBP: Systolic blood pressure
T2D: Type 2 diabetes
TMAO: Trimethylamine N-oxide
TG: Triglyceride
WHR: Waist-hip ratio
