# Supplementary material for: Population study of the gut microbiome: associations with diet, lifestyle, and cardiometabolic disease
Source: Genome Med. 2021 Dec 17;13:188. doi: 10.1186/s13073-021-01007-5 (PMC8680346; doi:10.1186/s13073-021-01007-5)
Supplement: Supplementary file 1 — Additional file 1. Supplementary figures. This file (.pdf) contains Figures S1-S3. [file 13073_2021_1007_MOESM1_ESM.pdf]

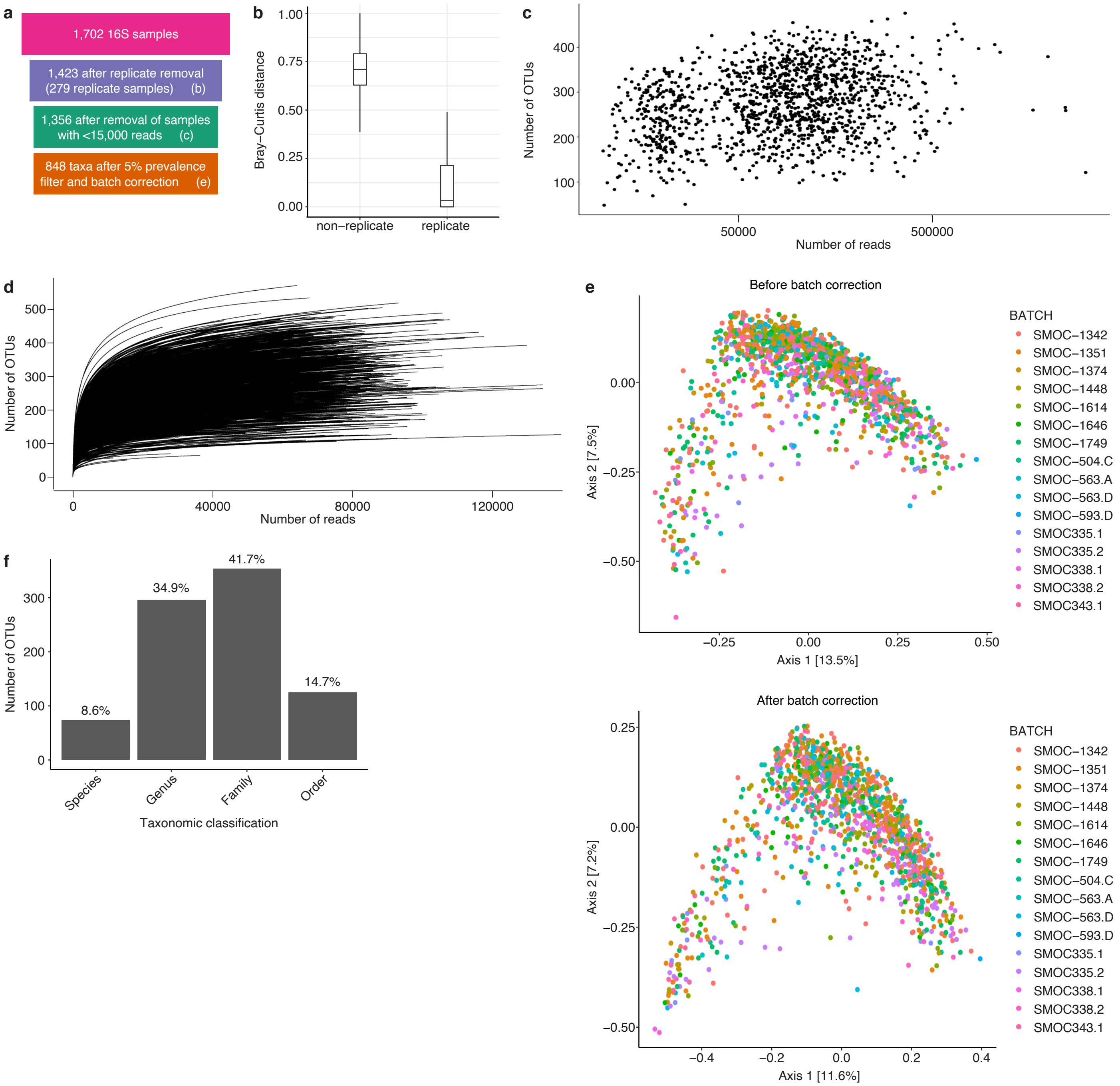

Figure S1. Quality control of 16S sequencing data. a) A sequential schematic of sample and OTU filtering steps. **b)** A boxplot of Bray-Curtis distances calculated from OTU-level relative abundances between replicate and non-replicate samples. **c)** A scatter plot of the number of reads per sample (after removal of samples with less than 15,000 reads) compared to the number of OTUs detected in that sample. **d)** Rarefication curves where each line represents one sample, showing the number of OTUs detected (y-axis) when read depth is downsampled to the number of reads (x-axis). **e)** Ordination plots of relative OTU abundance colored by sequencing batch before and after batch correction by MMUPHin. **f)** A histogram of the level of taxonomic classification achieved for each OTU.

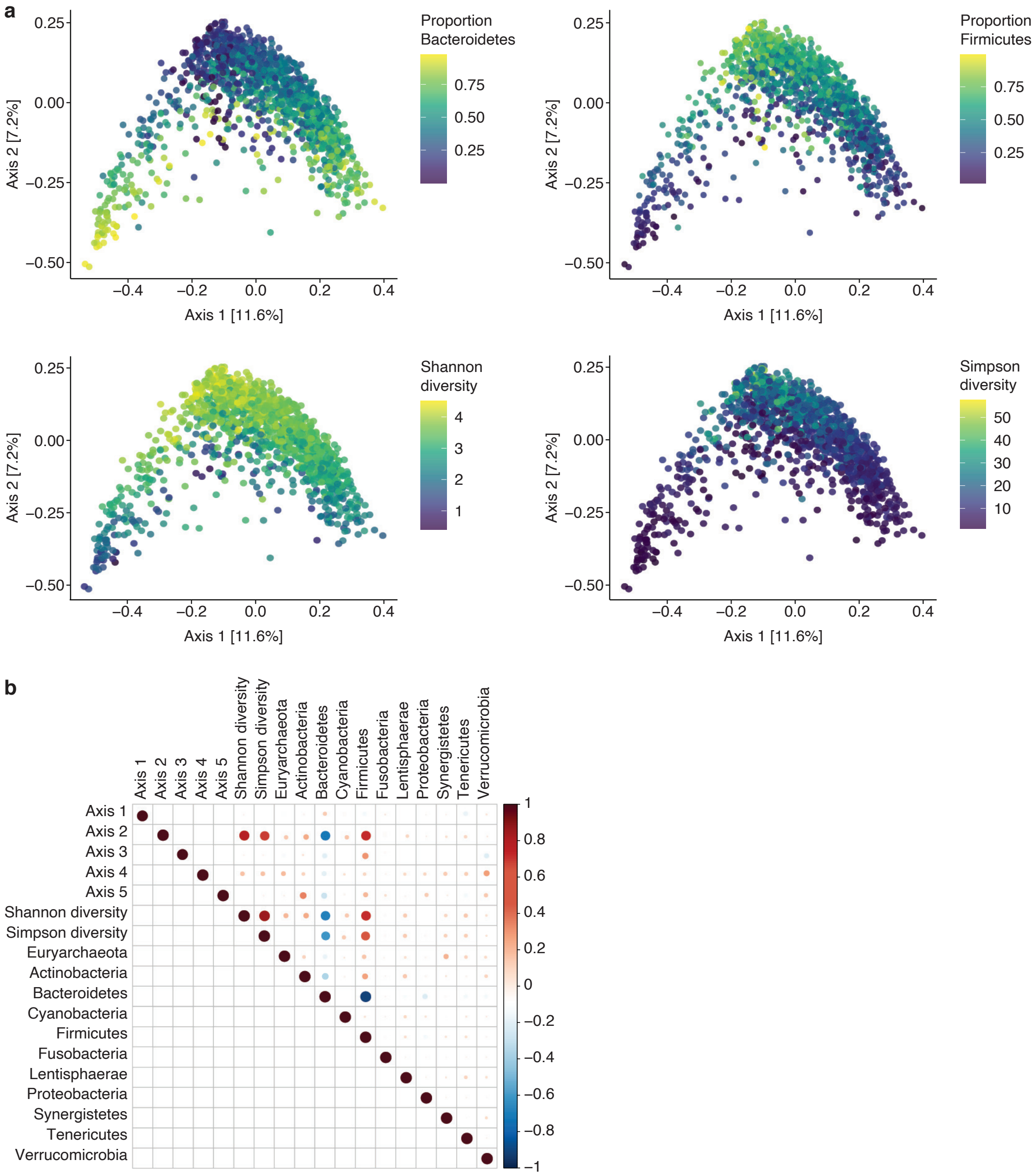

Figure S2. Multidimensional scaling plots in relation to phylum-level measurements. a) Multidimensional scaling (MDS/PCoA) analysis of Bray-Curtis distances calculated from OTU-level relative abundances. The two top axes of variation are shown on the x- and y-axis and respectively explain 11.6% and 7.2% of variation. Each dot represents an individual and is colored by the proportion of Bacteroidetes abundance, the proportion of Firmicutes abundance, Shannon diversity, and Simpson diversity. **b)** Pairwise correlations (Pearson) across the top 5 MDS axes from (a), Shannon diveristy, Simpson diversity, and all phylum-level relative abundances across individuals. Strength of correlation is shown by both the size and color of the dot. Color additionally shows the direction of correlation.

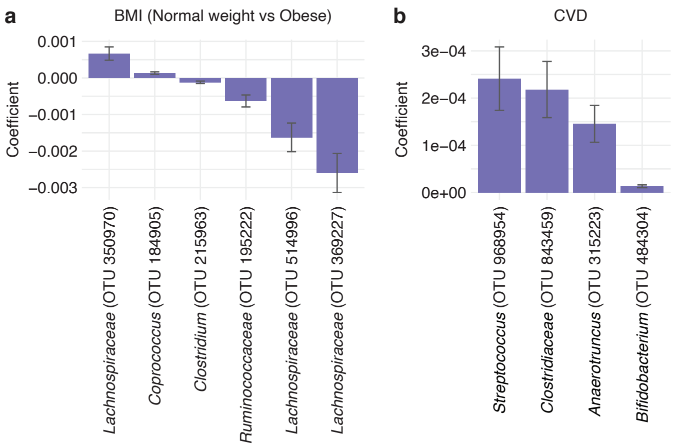

Figure S3. Effect sizes of top microbial associations with BMI and CVD. a) Bar plot of the coefficients from the linear model for significant OTUs associated with BMI status of normal weight versus obese participants. Standard error is shown. **b)** Bar plot of the coefficients from the linear model for significant OTUs associated with CVD status. Standard error is shown.
